# Emotional reactions towards COVID-19 among persons with diabetes

**DOI:** 10.1093/inthealth/ihab024

**Published:** 2021-05-11

**Authors:** Shiri Shinan-Altman, Inbar Levkovich

**Affiliations:** Louis and Gabi Weisfeld School of Social Work, Bar Ilan University, Ramat-Gan 52900, Israel; Faculty of Graduate Studies, Oranim Academic College of Education, Israel

**Keywords:** COVID-19, diabetes, emotional reactions, knowledge about COVID-19, perceived susceptibility, sense of mastery

## Abstract

The emotional impact of the coronavirus disease 2019 (COVID-19) pandemic on populations at large is emerging in the literature. However, the emotional response of persons with diabetes to the pandemic is only now beginning to emerge. Therefore this study aimed to identify factors contributing to emotional reactions towards this pandemic among persons with diabetes. A total of 205 persons with diabetes participated in this cross-sectional online survey between 14 May and 22 June 2020. Participants completed measures of emotional reactions towards COVID-19, perceived diabetes status, perceived susceptibility, knowledge about COVID-19, sense of mastery and sociodemographic questionnaires. Statistical analyses included Pearson correlations and regression analysis. According to the results, the mean score of negative emotional reactions towards COVID-19 was 3.45 (standard deviation 1.11, range 1–5), meaning that the score was relatively high. Higher levels of negative emotional reactions towards COVID-19 were associated with higher perceived susceptibility, greater knowledge about COVID-19 and a lower sense of mastery. The study's findings emphasize the need to communicate ongoing knowledge regarding COVID-19 and diabetes as well as to provide persons with diabetes with the necessary emotional support related to coping with diabetes and COVID-19.

## Introduction

Since November 2019, coronavirus disease 2019 (COVID-19) has progressively spread globally and aroused a wave of public health concerns.^1^ Accordingly, on 30 January 2020, the World Health Organization (WHO) declared the COVID-19 outbreak a Public Health Emergency of International Concern, and on 11 March the epidemic was upgraded to the level of a pandemic.^2^ In Israel, the first person with COVID-19 was diagnosed on 21 February 2020. Since then, thousands have been diagnosed with this disease and, as of 27 December 2020, 3225 people have died from the disease.^3^

Another long-standing global epidemic is diabetes, which was reported to affect 463 million adults (20–79 y of age) worldwide in 2019, which accounts for 9.3% of the world's population in this age group.^4^ In Israel, it is estimated that 415 800 adults have diabetes, or 8.1% of the adult population.^5^ People with diabetes are at a higher risk to develop complications when infected by a virus, such as COVID-19.^6^ Diabetes was found to be associated with mortality, severity and acute respiratory distress syndrome in COVID-19.^7^ However, in addition to the physical health implications that COVID-19 has on persons with diabetes, this virus also has emotional implications on this unique group.^8^ Indeed, studies have already shown a higher relationship between diabetes and a variety of emotional states (e.g. depression, anxiety),^9,10^ which could easily be exacerbated in a stressful environment, such as that surrounding everything related to COVID-19.^11^ Although the knowledge base regarding the responses of persons with diabetes to trauma and adverse events, in general, has been expanding,^12,13^ descriptions of their emotional responses during epidemics remain scarce. Yet their vulnerability makes this an important group to study. Given all of the above, this study aimed to explore emotional reactions towards COVID-19 among persons with diabetes in Israel during the COVID-19 pandemic. In addition, we explored several COVID-19-related factors and a psychological factor that may be associated with emotional reactions towards COVID-19 among persons with diabetes.

The proposed factors stem from the stress, appraisal and coping model.^14^ According to this model, a state of duress is a dynamic state of imbalance between oneself and one's surroundings, when the latter are perceived as placing demands on one's personal well-being. Specifically, in this study we explored COVID-19-related factors, namely knowledge regarding COVID-19 and perceived susceptibility of contracting COVID-19, and also sense of mastery.

Overall, knowledge of infection pathways and the relevant precautions one should take is needed to control the pandemic.^15^ However, people receive their information about COVID-19 from different sources and thus may have different knowledge about COVID-19, which may affect their emotional responses.^16^ The construct of perceived susceptibility has been used to measure one's perception of the likelihood of experiencing a virus or a disease.^17^ A study conducted in China among 585 people diagnosed with diabetes and 8431 who had not been diagnosed with diabetes found that people with diabetes perceived themselves to be at higher risk of COVID-19 infection and experienced higher levels of worry compared with those without diabetes.^8^

Sense of mastery, or the control belief, is defined as a powerful psychological resource connected to positive physical and mental states in prolonged stressful situations.^18^ Sense of mastery has been recognized as an indicator of resilience,^19^ and people may demonstrate better abilities to manage unexpected situations and related adversity if they possess greater control over themselves.^20^ Furthermore, psychosocial factors, such as sense of mastery,^21^ are reported to protect against developing psychological distress. According to the stress, appraisal and coping model, perceptions of stressful events (e.g. perceived susceptibility) are associated with psychosocial factors (e.g. sense of mastery), while the latter play a mediating role between how the stressful events are perceived and their outcome.^14^

To sum up, the current study represents the emotional reactions towards COVID-19 among persons with diabetes during the COVID-19 outbreak. This study aims to identify factors contributing to emotional reactions towards this pandemic among persons with diabetes. Addressing this aim may assist healthcare professionals in safeguarding the psychological well-being of chronic patients, such as persons with diabetes, in the face of a virus outbreak. The study's hypothesis was that a combination of perceived diabetes status, perceived susceptibility, knowledge about COVID-19 and sense of mastery will be related to emotional reactions towards COVID-19 among persons with diabetes.

## Methods

### Procedure and participants

We adopted a cross-sectional survey design to assess the emotional responses persons with diabetes during the COVID-19 epidemic by using an anonymous online questionnaire. Bar-Ilan University's Ethics Committee approved the study (authorization 052002). To minimize personal contact during the outbreak, the questionnaires were administered via a Qualtrics online platform (www.qualtrics.com). Participants were recruited mainly via internet forums (Facebook, Instagram) and websites dealing with diabetes, as well as through social media outlets and referral (word of mouth by participants who had already completed the survey). The study was advertised by notes on these websites that contained a short explanation about the study and a link to the questionnaire. To preserve anonymity, participants were not asked to provide any identifying information. A total of 210 Israelis visited the online survey between 14 May 2020 and 22 June 2020. This period reflects the subsiding of the first wave of COVID-19 in Israel. During this time, many individuals were requested to take a leave of absence or worked from home. Toward the end of the survey, a new routine was established, including the mandatory wearing of face masks and strict social distancing measures. Inclusion criteria were persons who were diagnosed with diabetes, age ≥18 and Hebrew speakers. Exclusion criteria included responses in set (the same answer number throughout the questionnaire; n=5).

### Measures

#### Dependent variable

Emotional reactions towards COVID-19 were assessed based on previous studies conducted among the general public^15^ using three questions concerning stress, fear and worry as a result of the pandemic (e.g. ‘How much do you worry in relation to COVID-19?’). Answers were rated on a 5-point Likert-type scale, ranging from 1 (not at all) to 5 (very much). A composite index of the average of all items was created, with a higher score indicating higher levels of negative emotional reactions towards COVID-19. The index exhibited strong internal consistency (Cronbach's α=0.93).

#### Independent variables

Perceived susceptibility was assessed based on previous studies conducted among the general public,^15^ with a one-item measure examining how likely the participant thought it was that he/she would contract the virus. ‘How likely do you think it is that you will get COVID-19?’ Answers were rated on a 5-point Likert-type scale ranging from 1 (not at all likely) to 5 (very likely).

Knowledge about COVID-19 was measured using a six-item COVID-19 subjective knowledge test assessing the symptoms, diagnosis, risk factors, ways of contracting COVID-19, ways to protect oneself from contracting COVID-19 and knowledge regarding where to refer a person who is suspected of having COVID-19. The scale's validity was reached by expert validity, a form of content validity. In this validity process the scale is reviewed by a panel of four expert physicians in order to eliminate totally irrelevant items from the instrument^22^ and to rephrase or supply new wording for items related to the measured construct where necessary.^23^ Answers were rated on a 5-point Likert-type scale ranging from 1 (don't know at all) to 5 (know very much). A composite index of the average of all items was created, with a higher score indicating higher levels of knowledge about COVID-19. The index exhibited an acceptable internal consistency (Cronbach's α=0.78).

Sense of mastery was assessed with a Hebrew-validated version^24^ of the Personal Mastery Scale consisting of seven items measuring the ability to deal with or exert control over issues as they arise in people's lives.^18^ Participants were asked to indicate the extent to which they agreed or disagreed with each item on a 5-point Likert scale ranging from 1 (strongly disagree) to 5 (strongly agree) (e.g. ‘What happens to me in the future mostly depends on me’). The mean score was calculated; a high score indicated higher levels of sense of mastery. The index exhibited an acceptable internal consistency (Cronbach's α=0.78).

Sociodemographic variables included gender, age, years of education, marital status and number of children.

Health information included perceived diabetes status (good/moderate/severe; recoded into good=1 vs. moderate/severe=0), other chronic diseases (yes/no) and time since the initial diagnosis of diabetes.

### Statistical analyses

Data analysis was carried out using SPSS version 25 (IBM, Armonk, NY, USA). Means, standard deviations (SDs) and Pearson correlations were determined to assess the associations among the study variables. A multiple regression was conducted to predict emotional reactions towards COVID-19.

## Results

As can be seen in Table 1, the study included 205 persons with diabetes, most of whom were female. The mean age was 40.18 y (SD 15.52) and the mean number of years of education was 15.07 (SD 3.40). About half of the participants were married and the mean number of children was 2.49 (SD 0.91). In addition, the mean time since initial diagnosis of diabetes was 15.70 y (SD 12.47). More than half of the participants perceived their diabetes as being moderate to severe and 42.9% had additional diseases. Among these additional diseases were hypertension (41.4%), coronary heart disease (15.7%) and other diseases (25.7%).

**Table 1.** Participants’ characteristics (N=205)

Characteristics	Values
Gender, n (%)	
Male	40 (20.8)
Female	152 (79.2)
Age (years), mean (SD), range	40.18 (15.52), 18–80
Years of education, mean (SD), range	15.07 (3.40), 9–35
Marital status, n (%)	
Married	106 (51.7)
Not married	99 (48.3)
Number of children, mean (SD), range	1.45 (1.41), 0–5
Time since initial diagnosis of diabetes (years), mean (SD), range	15.70 (12.47), 1–54
Subjective health status, n (%)	
Moderate–severe	127 (63.2)
Good	74 (36.8)
Other chronic diseases, n (%)	
Yes	87 (42.9)
No	116 (57.1)

Table 2 summarizes the means, SDs, ranges and correlates of the study's variables. The mean score of emotional reactions towards COVID-19 was 3.45 (SD 1.11, range 1–5), meaning that the score was relatively high. The mean score of perceived diabetes status was 0.37 (SD 0.48, range 0–1), the mean score of perceived susceptibility was 2.62 (SD 0.86, range 1–5) and the mean score of sense of mastery was 2.82 (SD 0.46, range 1–5). All three of these scores were relatively high.

**Table 2.** Correlates, means, SDs and ranges of study variables (N=205)

Variables	1	2	3	4	5
1. Emotional reactions towards COVID-19	–				
2. Perceived diabetes status	−0.24^**^	–			
3. Perceived susceptibility	0.37^**^	−0.28^**^	–		
4. Knowledge about COVID-19	0.20^**^	−0.13	−0.05	–	
5. Sense of mastery	−0.31^**^	−0.13	−0.14*	0.03	–
Mean	3.45	0.37	2.62	4.00	2.82
SD	1.11	0.48	0.86	0.56	0.46
Possible range	1–5	0–1	1–5	1–5	1–5
Actual range	1–5	0–1	1–5	2.67–5.00	1–5

* p<0.05, ^**^p<0.01, ^***^p<0.001

According to Table 2, significant associations were found linking emotional reactions towards COVID-19 with perceived diabetes status, perceived susceptibility, knowledge about COVID-19 and sense of mastery. This means that the more participants had negative emotional reactions towards COVID-19, the more they were likely to perceive their diabetes status as being moderate to severe, had higher perceived susceptibility, higher knowledge about COVID-19 and a lower sense of mastery. Furthermore, perceived diabetes status was negatively associated with perceived susceptibility. In other words, participants who perceived their diabetes status as being moderate to severe reported lower levels of perceived susceptibility. Sense of mastery was positively associated with perceived susceptibility, meaning that the more participants reported a high sense of mastery, the less they perceived themselves to be at a high risk of contracting COVID-19.

In addition, emotional reactions towards COVID-19 were significantly higher among women (mean 3.60 [SD 1.09]) than men (mean 2.95 [SD 1.28]) (t(188)=−3.37, p=0.001) and was positively related to time since the initial diagnosis of diabetes (r=0.19, p=0.002). Emotional reactions towards COVID-19 were not associated with the number of children (r=0.44, p=0.53), age (r=0.45, p=0.563), years of education (r=0.11, p=0.10) or having additional chronic diseases (r=−0.11, p=0.12).

### Regression analysis for emotional reactions towards COVID-19

In light of the associations described above, the regression for emotional reactions towards COVID-19 was calculated while controlling for gender and time since the initial diagnosis of diabetes. The results in Table 3 show that the regression model for emotional reactions towards COVID-19 is significant, explaining 24.5% of the variance. It appears that a higher level of negative emotional reactions towards COVID-19 was associated with higher perceived susceptibility, higher knowledge about COVID-19 and a lower sense of mastery.

**Table 3.** Multiple regression for prediction of emotional reactions towards COVID-19 (N=156)

	Emotional reactions towards COVID-19		
Independent variables	B	SEB	β
Gender	0.32	0.18	0.13
Time since initial diagnosis of diabetes	0.12	0.00	0.14
Perceived diabetes status	−0.09	0.16	−0.04
Perceived susceptibility	0.35	0.09	0.30^***^
Knowledge about COVID-19	0.31	0.13	0.17*
Sense of mastery	−0.51	0.16	−0.27^**^
R^2^ (adjusted R^2^)	0.245^***^		
F(df)	9.41 (6150)		

SEB: standard error of B; df: degrees of freedom.

* p<0.05, ^**^p<0.01, ^***^p<0.001.

The regression analysis did not include participants who did not complete the questionnaire in its entirety.

## Discussion

The goal of the current study was to explore emotional reactions towards COVID-19 among persons with diabetes in Israel during the COVID-19 pandemic outbreak. We also explored several COVID-19-related factors that may be associated with emotional reactions towards COVID-19 among persons with diabetes.

Our findings suggest that persons with diabetes experience relatively higher negative emotions towards COVID-19. This finding is in line with those of previous studies, which documented higher negative emotions towards COVID-19 among the general population^15,25^ and among people with chronic illness, such as breast cancer patients.^26^ Overall, the existing chronic condition of diabetics may place them at a greater risk for developing more complications as a result of COVID-19,^2^ which can increase reactions of worry, fear and stress and ultimately exacerbate their health and well-being.^27^ These days, most international media attention is focused on the direct causes of COVID-19; however, it is also important to address the negative emotional reactions resulting from COVID-19, especially among vulnerable populations.

Our results indicate that perceived susceptibility was associated with higher levels of negative emotional reactions towards COVID-19. This finding is in line with the assumptions of the Self-Regulation Model, which claims that the way one perceives a health threat is associated with his/her emotional reactions.^28^ An increase in generalized fear was also found during other outbreaks, such as severe acute respiratory syndrome in 2003 and the Ebola virus in 2014.^29^ These emotional responses were particularly high among high-risk persons.^29^ Overall it should be noted that during the COVID-19 outbreak, the media has provided ongoing information about the virus while addressing at-risk groups, including persons with diabetes. Although information about the virus and its characteristics has varied over time, one of the constant messages provided by both the Israeli media and the global media is that people with chronic diseases, especially people with diabetes, need to pay particular attention to preventative behaviours, as they are a vulnerable population.^2,3^ It is possible that these consistent messages regarding their high-risk status influence the self-perceptions of persons with diabetes about being at risk—and, in the end, elicit negative emotional responses.

In this study, a lower sense of mastery was associated with higher negative emotions towards COVID-19. This finding is in line with another study conducted during the COVID-19 outbreak that found psychological factors, such as a sense of control, were associated with less psychological distress.^30^ Accordingly, providing precise and clear information regarding measures that enhance individuals’ perceived control over a threat may limit negative emotional reactions.^31,32^ The present finding, which indicates that a psychological factor such as sense of mastery is related to the emotions one feels towards COVID-19, underscores the importance of further research examining the effects of psychological factors over time during a pandemic crisis. Indeed, psychological factors may change over time, thus influencing the emotional reactions among persons with diabetes.

This study has several limitations. First, given that the data presented here were derived from a cross-sectional design, it is hard to make causal inferences. Second, the study was limited to the time of the COVID-19 outbreak, therefore the sampling was voluntary and conducted via an online system. Accordingly, the possibility of selection bias should be considered. Third, the use of a convenience sample does not allow for generalizability, nor does it provide an accurate representation of all persons with diabetes in Israel.

Despite these limitations, our study is one of the first studies to identify factors contributing to emotional reactions towards COVID-19 among persons with diabetes. In addition, the findings of the current study expand the limited body of existing knowledge describing the effect of COVID-19 on persons with diabetes. This is especially important given that most of the current available data on persons with diabetes during COVID-19 are concentrated on diagnosis, treatment and health services utilization (e.g. ^33,34^). Practically, the findings present a mechanism for recognizing persons with diabetes with higher negative emotions towards COVID-19, namely, those who have higher perceived susceptibility, higher knowledge about COVID-19 and a lower sense of mastery. Accordingly, the study's findings emphasize the need to communicate all new information regarding COVID-19 and diabetes as well as to provide emotional support and guidance associated with emotional reactions related to diabetes and COVID-19.^35^ More knowledge about how to provide this support systematically is needed. It should be noted that even under normal circumstances, it is often not possible for persons with diabetes to access emotional support. In the case of the COVID-19 pandemic, this access may prove even more difficult to achieve, despite its increased importance.^36^ Nevertheless, our findings suggest that intervention programs may benefit from increasing a sense of mastery among persons with diabetes, as this may improve diabetics’ coping and may decrease negative emotional responses during a pandemic outbreak. Finally, given the importance of sense of mastery among persons with diabetes, it is suggested to examine the mediating role of sense of mastery on the link between perceived susceptibility and emotional reactions towards COVID-19 in future studies.

## Data Availability

The data that support the findings of this study are available from the authors upon reasonable request.
